# From Plants to Psycho-Neurology: Unravelling the Therapeutic Benefits of Bioactive Compounds in Brain Disorders

**DOI:** 10.3390/antiox12081603

**Published:** 2023-08-11

**Authors:** Clara Grosso, Marlene Santos, M. Fátima Barroso

**Affiliations:** 1 REQUIMTE–LAQV, Instituto Superior de Engenharia do Porto, Instituto Politécnico do Porto, Rua Dr. António Bernardino de Almeida 431, 4249-015 Porto, Portugal; claragrosso@graq.isep.ipp.pt; 2 CISA|ESS, Centro de Investigação em Saúde e Ambiente, Escola Superior de Saúde, Polytechnic Institute of Porto, Rua Dr. António Bernardino de Almeida 400, 4200-072 Porto, Portugal; mes@ess.ipp.pt

**Keywords:** oxidative stress, psychiatric disorders, neurodevelopmental disorders, neurodegenerative disorders, phenolic compounds, plant-derived bioactive compounds, antioxidant activity

## Abstract

The brain’s sensitivity to oxidative stress and neuronal cell death requires effective pharmacotherapy approaches. Current pharmacological therapies are frequently ineffective and display negative side effects. Bioactive chemicals found in plants may provide a potential alternative due to their antioxidant and neuroprotective properties and can be used in therapy and the management of a variety of neuropsychiatric, neurodevelopmental, and neurodegenerative illnesses. Several natural products, including vitamin C, *Cammelia sinensis* polyphenols, *Hypericum perforatum*, and *Crocus sativus* have shown promise in lowering oxidative stress and treating symptoms of major depressive disorder (MDD). Similarly, bioactive compounds such as curcumin, luteolin, resveratrol, quercetin, and plants like *Acorus gramineus*, *Rhodiola rosea*, and *Ginkgo biloba* are associated with neuroprotective effects and symptom improvement in neurodevelopmental disorders such as autism spectrum disorder (ASD) and attention deficit/hyperactivity disorder (ADHD). Furthermore, in neurodegenerative diseases, natural compounds from *Rhodiola rosea*, *Morinda lucida*, and *Glutinous rehmannia* provide neurological improvement. Further study in clinical samples is required to thoroughly investigate the therapeutic advantages of these bioactive substances for persons suffering from these illnesses.

## 1. Introduction

A biological antioxidant, according to Laguerre et al. (2010), is a molecule that, when present in low amounts compared to an oxidizable substrate, protects that substrate from oxidation (both directly and through its oxidation products), thus protecting the body from the damaging effects of oxidative stress [1]. In this context, oxidative stress arises when oxidants, namely reactive oxygen/nitrogen species (ROS/RNS) produced in cells through a range of enzymatic and non-enzymatic pathways, surpass the cell’s antioxidant capacity [2]. This imbalance causes molecular damage (such as in nucleic acids, lipids, and proteins), which has been linked to more than 100 disorders, including cardiovascular disease, cancer, and metabolic, psychiatric, neurodegenerative, and neurodevelopmental diseases [3].

Fortunately, nature has evolved complex antioxidant mechanisms to counteract and avoid the harmful effects of oxidants and to reduce oxidative stress in most living organisms. Endogenous antioxidants are enzymes or small molecules that are produced by the human body to repair oxidatively damaged spots on macrobiomolecules [4,5]. However, the human body’s inherent antioxidant defence systems are insufficient, and it requires a constant demand for exogenous antioxidant sources to avoid and regulate oxidative stress [6].

**Figure 1:**
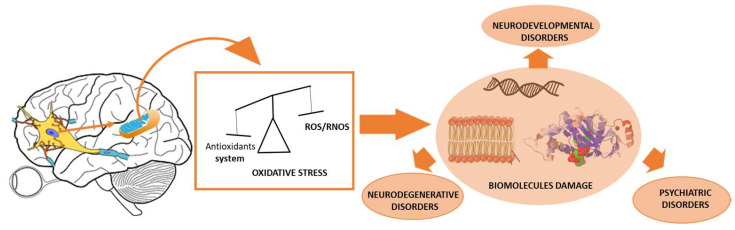
Association of neurodegenerative, neurodevelopmental, and psychiatric disorders to the imbalanced antioxidant/oxidant (ROS/FNOS) system.

Food, namely fruits and vegetables, is a good exogenous source of natural antioxidants. As a result, it is plausible that increasing dietary antioxidant consumption may aid in maintaining an appropriate antioxidant state and, therefore, the normal physiological functioning of a living system [4]. Even with a well-balanced diet, oxidative stress can emerge because of factors such as age and family history, alcohol and smoking, poor health, sleep issues, diabetes, cardiovascular health, and environmental risk factors (Figure 1) [7].

Plants, or more precisely, plant-isolated bioactive compounds, are being used to lower oxidative stress and to aid the treatment of a range of disorders as their health-beneficial qualities become more widely acknowledged. Furthermore, this aim is more applicable to disorders that are caused by or are impacted by oxidative stress [8,9,10,11].

This review paper highlights the potential of plant-derived bioactive compounds to be used with a therapeutical purpose in treatment of psychiatric, neurodevelopmental, and neurodegenerative disorders. An extensive literature search was conducted using prominent databases, including PubMED, Web of Science, Scopus, and Science Direct. The following keywords were used to identify relevant studies: “plants”, “bioactive compounds”, “psychiatric disorders”, “neurodegenerative disorders”, “neurodevelopmental disorders”, “Major depression disorder”, “bipolar disorder”, “schizophrenia spectrum disorder”, “autism spectrum disorder”, “attention deficit hyperactivity disorder”, “Alzheimer’s disease”, “Parkinson’s disease”, “neuroprotection”, and “therapy”. Only publications written in English were considered, and conference abstracts, book chapters, or books were excluded from the selection process. A total of 66 articles were identified, focusing on the relationship between bioactive compounds and brain disorders.

## 2. Bioactive Compounds from Plants as Antioxidant, Anti-Inflammatory, and Neuroprotective Agents

**Figure 2:**
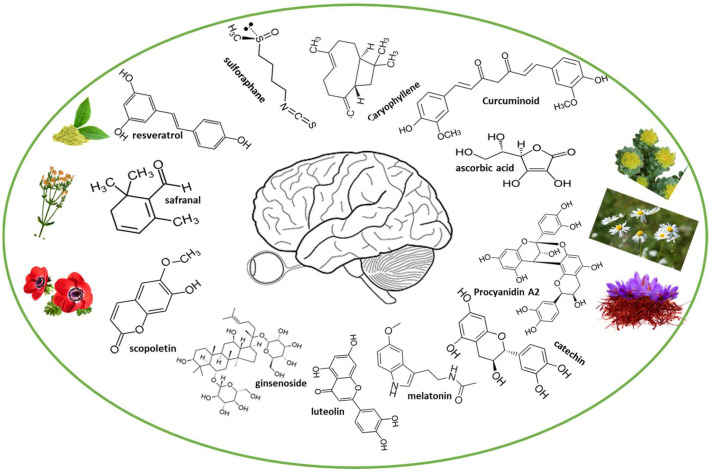
Plant-derived bioactive compounds for brain neuroprotection.

The brain is an organ especially sensitive to oxidative damage, due its high and specific metabolic activity. High consumption of oxygen (which induces the generation of ROS and RNS), almost solely oxidative phosphorylation, lack of energy stores, high quantities of lipids susceptible to peroxidation, and high iron levels all operate as pro-oxidant molecules. Because of this, oxidative stress and related metabolic/ischemic damage to neuronal cells are of a serious concern [12]. Thus, these factors may explain the ROS/RNS involvement in several neuropsychiatric, neurodevelopmental, and neurodegenerative disorders. An antioxidant diet is known to reduce oxidative stress, while a pro-oxidant diet increases the probability of having health problems. The current focus of attention lies in exploring the protective and therapeutic potential of bioactive compounds derived from recognized plants, which exhibit antioxidant, anti-inflammatory, and neuroprotective properties (Figure 2).

### 2.1. Neuropsychiatric Disorders

According to the DSM-5 (Diagnostic and Statistical Manual of Mental Disorders), a psychiatric disorder (PD) is a syndrome characterized by clinically significant disturbance in an individual’s cognition, emotion regulation, or behaviour that reflets a dysfunction in the psychological, biological, or developmental processes underlying mental functioning. PDs are usually associated with significant distress or disability in social, occupational, or other activities [13]. According to the World Health Organization (WHO), PDs are, at this moment, the second leading cause of disability worldwide [14]. Some of the most common PDs include depression, schizophrenia spectrum (SZ), and bipolar disorder (BD) [15].

#### 2.1.1. Major Depression Disorder: Plant-Based Bioactive Compounds as Therapeutical Agents

Major depression disorder (MDD) is a multifaceted and heterogenous mood disorder with a complex etiopathogenesis. MDD is illustrated by permanent feelings of sadness, hopelessness, and loss of interest in activities accompanied by a range of other physical and cognitive symptoms, such as changes in appetite or sleep, fatigue, difficulty concentrating, and feelings [16]. Unfortunately, the World Health Organization (WHO) estimates that 280 million people suffer from depression worldwide [17]. Antidepressants are the main pharmacological treatment for MDD. Selective serotonin reuptake inhibitors (SSRIs), serotonin–norepinephrine reuptake inhibitors (SRNIs), tricyclic antidepressants (TCAs), MAO-A inhibitors (MAO-AIs), and other drugs like bupropion and mirtazapine are among the main antidepressants used nowadays [18]. Despite the effectiveness and overall tolerability of current antidepressant medications, a significant proportion of patients (ranging from 23% to 44%) experience adverse drug reactions, leading to treatment discontinuation. Additionally, a substantial number of patients (ranging from 20% to 40%) demonstrate minimal response to monotherapy [19].

So, there is a growing need to find new and more effective drugs that can be used with patients exhibiting a lack of response. Therefore, the discovery of plant-derived antioxidant compounds that might lessen depression is greatly desired.

Ascorbic acid, often known as vitamin C, is one powerful antioxidant and an essential vitamin in the human diet. Citrus fruits, berries, tomatoes, potatoes, and green leafy vegetables are excellent sources of vitamin C. There is evidence that vitamin C levels are not equally distributed in the human body, with the brain having a higher concentration than the periphery. As a result, disorders linked with low plasma ascorbic acid concentrations are mostly connected to the central nervous system [20]. Furthermore, vitamin C has been shown to influence neurotransmission by inhibiting neurotransmitter binding to receptors, altering neurotransmitter release and reuptake, or acting as a cofactor for neurotransmitter synthesis. In addition, vitamin C stimulates synaptic release of acetylcholine (ACh) and norepinephrine, connecting vitamin C to promote neurotransmission [20].

The tea plant (*Camelia sinensis* (L.) Kuntze) contains an abundance of bioactive compounds with antidepressant properties [21]. Horia et al. [22] demonstrated that many different tea-derived polyphenols, namely flavan-3-ols (catechin, gallocatechin, epicatechin), flavonols (kaempferol, quercetin), and phenolic acids (gallic and chlorogenic acid), presented anti-inflammatory activity via down-regulation of nuclear factor kappa-light-chain-enhancer of activated B cells’ (NF-κB) signalling pathway and antioxidant activity, which can be vital to reduce the risk of depression [21,22]. An important preclinical study demonstrated a significant improvement in memory and cognition in animal models of neurodegenerative diseases. Phenolic consumption has been associated with increased cognitive function, and their administration may prevent cognitive decline [22].

*Hypericum perforatum* L. (St. John’s wort) is another well-studied Chinese medicinal plant whose bioactive compounds (hyperforin, rutin, and melatonin) have been reported to have anti-inflammatory, antioxidant, antifatigue, and antidepressant effects [23]. Similarly, a closely related species, *Hypericum triquetrifolium* Turra, also presents several bioactive compounds, namely hypericin. This compound is an antioxidant naphthodianthrone which is also probably responsible for the increased hippocampal brain-derived neurotrophic factor (BDNF) concentration induced by *H. triquetrifolium* administration, thus reverting the depression and stress-induced cognitive deficit [24].

The spice saffron, which is extracted from the floral stigmas of *Crocus sativus* L. and used as a culinary ingredient, has in its composition several bioactive compounds with anti-inflammatory and antioxidant effects. These may include curcumin, crocins, crocetin, picrocrocin, and safranal. The molecular mechanism action of turmeric curcumin and curcumin is based on the inhibition of the NOD-like receptor protein (NLR3) inflammasome and by the regulation of the tryptophan amino acid degradation products (kynurenine and quinolinic acid), which present neuroprotective effects. Furthermore, curcumin may also reduce oxidative stress by lowering the oxidative stress marker malondialdehyde [25,26]. Likewise, another study indicated that curcumin can also reduce the ROS levels by increasing the production of antioxidants that promote the increase in the transcription factor of nuclear factor erythroid 2-related factor 2 (NRF2) [27,28]. In addition, results from clinical studies, analysing the effect of curcumin versus a placebo, showed clinically significant efficacy of curcumin in reducing depressive symptoms. Table 1 shows the most relevant plant-derived bioactive compounds with neuroprotective actions.

#### 2.1.2. Bipolar Disorder: Plant-Based Bioactive Compounds as Therapeutical Agents

Bipolar disorder (BD) is a chronic mood disorder characterized by manic or hypomanic episodes alternating or intermixed with episodes of depression with a prevalence of 1% of the world population [29]. BD is classified into two categories: type I (episodes of depression and persistent mania) and type II (episodes of depression and hypomania) [13]. The exact pathophysiology of the disease has not yet been determined, but more than 85% of cases are due to heredity [30]. Nevertheless, it has been discovered that in BD individuals, there is a relative overlap of the catechol-o-methyltransferase (COMT) gene which controls dopamine metabolism [30]. Oxidative stress is one of the major factors described in the aetiology of mania; therefore, bioactive compounds that display antioxidant activity and eliminate oxidative stress are good candidates as an anti-manic agent.

Ethanolic extracts of saffron (*Crocus sativus* L.) and its constituents safranal (monoterpenoid) and crocin (carotenoid) have been used in preclinical animal models and have shown antidepressant effects. *Curcuma longa* L. and *H. perforatum* are other plants used in various nervous system disorders and have been used over the past decades in the treatment of MDD [31]. Carvone, a monoterpene present in volatile oils of *Mentha* spp. and *Carum carvi* L., resulted in decreased locomotor activity in the tested animals, probably due to the GABAergic activity and sodium channel block [32].

The hydroxybenzoic acid gallic acid, widely produced in plants, was used in the treatment of ketamine-induced mania in rats and compared to the action of lithium. It was observed that gallic acid decreased the hyperlocomotion of the animals, induced antioxidant properties, and prevented cholinergic disfunctions in the brain [33]. Moreover, quercetin, a flavonoid, also showed antioxidant properties and inhibition of protein kinase C [34]. Table 2 shows the most relevant plant-derived bioactive compounds with neuroprotective effects in BD patients.

#### 2.1.3. Schizophrenia Spectrum Disorders: Plant-Based Bioactive Compounds as Therapeutic Agents

Around 1–1.5% of the world population suffer from schizophrenia (SZ). SZ is defined by abnormalities in one or more of the following five domains: delusions, hallucinations, disorganized thinking, grossly disorganized or abnormal motor behaviour and negative symptoms [35]. Abnormalities in production of neurotransmitters such as dopamine, serotonin, glutamate, aspartate, glycine, and gamma-aminobutyric acid (GABA) are the most major pathophysiological cause of SZ [36]. Recently, it was verified that some natural bioactive compounds, namely the terpenoids beta-caryophyllene, limonene, saponin, and polygalasaponin, have potential antipsychotic by promoting the cannabinoid receptors inhibition [37].

Mitra et al. [38] found out that scopoletin, a coumarin present in the root of *Scopolia carniolica* Jacq. and *Scopolia japonica* Maxim., acts as an antidopaminergic agent potentially because of the stimulation of the epidermal growth factor receptor (ErbB1 and ErbB2) phosphorylation process, alleviating the symptoms of the schizophrenia psychosis [38]. Likewise, the anthraquinone emodin isolated from *Reynoutria japonica* Houtt. (syn. *Fallopia japonica* (Houtt.) Ronse Decr.) also acts as an antipsychotic agent by increasing the phosphorylation process and the ErB1 and ErbB2 levels [38]. The phenolic compound curcumin extracted from *C. longa* impacted the BDNF levels leading to a decrease in Il-6 levels [39]. Thirty-eight individuals with persistent schizophrenia were studied to determine the effectiveness of curcumin as an additional agent to standard antipsychotic drugs. In this clinical experiment, patients received antipsychotic medication and 3000 mg/day of curcumin, or a placebo, for 24 weeks. Curcumin’s encouraging results in treating negative symptoms when combined with antipsychotics may create a new, secure therapeutic alternative for the treatment of schizophrenia [39]. Table 3 shows the most relevant plant-derived bioactive compounds with neuroprotective effects in SZ patients.

### 2.2. Neurodevelopmental Disorders

According to the diagnostic and statistical manual of mental disorders (DSM-5), “neurodevelopmental disorders are a group of conditions with onset in the developmental period. The disorders typically manifest early in development, often before the child enters grade school, and are characterized by developmental deficits that produce impairments of personal, social, academic, or occupational functioning. The range of developmental deficits varies from very specific limitations of learning or control of executive functions to global impairments of social skills or intelligence” [13]. Some of the most common neurodevelopmental disorders include autism spectrum disorder (ASD) and attention deficit/hyperactivity disorder (ADHD) [29].

#### 2.2.1. Autism Spectrum Disorder: Plant-Based Bioactive Compounds as Therapeutic Agents

ASD, with a prevalence of 1% over the world, encompasses a heterogenous continuum of neurodevelopmental disorders by a range of social and communication deficit severities coupled with repetitive and unusual sensory–motor behaviour deficits in social exchange and communication, as well as fixed interests and stereotypic repetitive behaviours [40]. Recently, the mitochondrial dysfunction, namely oxidative stress, has been associated to the aetiology of ASD. In fact, the ASD postmortem brain samples analysis indicated the presence of brain mitochondrial dysfunction proved by the existence of discrepancies in gene expression, a decrease in the electron transport chain activity as well as a decrease in the tricarboxylic acid cycle enzyme [41]. Furthermore, the contribution of oxidative stress was also observed as one of the causes of this disorder [42]. So, it not surprising that several studies found a connection between dietary deficiencies and ASD, as a consequence. To help ASD individuals, several plant-based bioactive compounds have been studied as a complement to ASD therapy [42,43].

Curcumin has been mentioned several times as a potential neuroprotector in several disorders belonging to psychiatric, neurodevelopmental, and neurodegenerative systems. Curcumin in combination with propionic acid has been described to have the ability to reduce oxidative–nitrosative stress, mitochondrial dysfunction, and tumour necrosis factor-α (TNF-α) and matrix metalloproteinase-9 (MMP-9) production [44]. Another study indicated that curcumin easily cross through the blood–brain barrier. In the brain, it can increase the GSH concentration, leading to a reduction in protein cluster, mitochondrial dysfunction, and oxidative stress, improving ASD life quality [45].

Vegetables and fruits such as broccoli, onion leaves, carrots, peppers, cabbages, and apple skins have high amounts of the flavonoid luteolin. Luteolin has been reported to have excellent neuroprotective effect by (i) reducing the activation of the immune neural abnormalities, the serum interleukin-6 and tumour necrosis factor-α concentration, and the nitrotyrosine and nuclear factor kappa B (nF-kB); (ii) improving the neuroplasticity and neurogenesis; and (iii) inhibiting the mast-cell activation by stimulating the mitochondria [46,47,48,49].

Resveratrol is a polyphenolic bioactive compound found in grapes, peanuts, cocoa, and berries. Like luteolin, resveratrol has been indicated to have exceptional neuroprotective action. As it has been described in the literature, resveratrol has the capacity to activate the adenosine 3′,5′-monophosphate (AMP)-dependent protein and to stimulate the sirtuin-1/peroxisome proliferator-activated receptor-γ coactivator-1α (SIRT1/PGC-1α)-dependent effect, which consequently increases mitochondria biogenesis [50]. Moreover, resveratrol also reduces TNF α expression, neuroinflammation, and oxidative stress, making it a promising choice for improving ASD neurobehavioural and biochemical alterations [51]. To explore the ameliorative potential of resveratrol on neuroinflammation, Bhandari and collaborators performed an experimental study using a neuroinflammatory model of ASD in rats. For that, propanoic acid was infused over 10 min into the anterior portion of the lateral ventricle to induce ASD-like symptoms in rats. Then, resveratrol (5, 10, and 15 mg/kg) was administered starting from the 2nd day of surgery and continued up to the 28th day. The major finding of this study is that resveratrol restored the core and associated symptoms of autistic phenotype [51].

Isothiocyanate sulforaphane, present in high amounts in broccoli sprouts, has been also reported the found to enhance ASD symptoms by increasing glutathione (GSH) production and by reducing oxidative phosphorylation, lipid peroxidation, and neuroinflammation [52].

Quercetin present in *Chamomile* sp., *Sophora* sp., and *C. sinensis* extracts is bioaccumulated in its active forms in the mitochondria organ, which promote the mitochondria protection by increasing the scavenging antioxidant activity against ROS generated in the cell [53,54]. Likewise, rutin also has neuroprotective effect against ASD by preventing the ATP drop and indomethacin-induced alteration in the mitochondrial membrane [51]. Table 4 shows the most relevant plant-derived bioactive compounds with neuroprotective effects in ASD patients.

#### 2.2.2. ADHD: Plant-Based Bioactive Compounds as Therapeutic Agents

ADHD is the most common neurodevelopmental disorder, affecting around 5% of children and adolescents and 2.5% of adults worldwide [55,56]. The core symptoms clusters of ADHD, defined as inattention and/or impulsivity–hyperactivity, are associated with problems in remaining focused on a task for prolonged periods, as well as difficulties in organizing activities, prioritizing tasks, and time management [13]. Studies involving neuroimaging indicated that one of the underlying causes of ADHD is the presence of abnormalities in the prefrontal and parietal brain area. Additionally, from a neurobiological and cellular point of view, clinical symptoms of ADHD have been associated with changes in the dopaminergic and noradrenergic system as well as with glutamatergic pyramidal neurons and glutamatergic neurotransmission [42,57].

Regarding ADHD treatment, a growing number of natural product formulations have become common with the primary objective of avoiding or even reducing the use of psychotropic drugs. Recently, the focus on plant-occurring bioactives for therapeutic purposes has increased. Several medicinal-based plants have been studied for therapeutical efficacy in ADHD, namely *Acorus gramineus* Aiton, *Rhodiola rosea* L., *Psoralea corylifolia* L., *H. perforatum* L., *Valeriana officinalis* L., *G. biloba* L., *Scutellaria baicalensis* Georgi, *Sideritis clandestina* (Bory and Chaub) Hayek, *Paeonia lactiflora* Pallas, *Withania somnifera* (L.) Dunal, *Centella asiatica* (L.) Urb., *Bacopa monnieri* (L.) Wettst., *Mellissa officinalis* L., and *Passiflora incarnata* L. being the most widely exploited for such purposes.

The phenylpropanoids α-asarone and β-asarone isolated from *A. gramineus* extracts have potential to reduce the acetylcholinesterase activity and to supress interleukin-(Il-6), IL-1β, nitric oxide synthase, and cyclooxygenase (COX)-2 expression, leading to an increase in cognition and synaptic plasticity [58]. On the other hand, Oroxylin A, a flavonoid isolated from the root of *S. baicalensis*, exhibits antioxidant, anti-inflammatory, memory-enhancing, and neuroprotective effects by inhibiting the modulation of gamma-aminobutyric acid (GABA)-A receptor and improving dopamine neurotransmission [59].

Alongside Oroxylin A, the flavonoid baicalin (from *S. baicalensis*) also presents antioxidant activity with positive effects in ADHD symptoms [60]. Methylphenidate, a drug used to treat ADHD, along with ginsenoside from ginseng extracts and terpenoids from *G. biloba* extracts, reduced the expression of Tropomyosin receptor kinase B (TrkB), BDNF, dopamine (DAT), and norepinephrine (NET) transporters [61].

Pycnogenol is a polyphenolic antioxidant compound extracted from the bark of the *Pinus pinaster* Aiton. This antioxidant significantly improves ADHD symptoms by decreasing the oxidative DNA damage and the dopamine concentrations and improving the GSH/GSSH ratio through the normalization of catecholamine concentration levels in children [62]. Likewise, curcumin can easily cross the blood–brain barrier, and induce neuroprotective activity by increasing the GSH levels and reducing oxidative stress and neuroinflammation [63]. The flavan-3-ols catechin and EGCG extracted from *C. sinensis* were shown to have anxiolytic and sedative effects improving ADHD symptoms due to their capacity to neuromodulated the dopamine and serotonin levels in some brain areas [64].

*H. perforatum* and its extracts have been widely used in traditional medicine. *H. perforatum* contains a wide range of chemical compounds including volatile oils, flavonoids, anthraquinone, prenylated phloroglucinols, tannins, and xanthones, among others. Nevertheless, the therapeutical important compounds in *Hypericum* species are the phloroglucinol hyperforin, the naphthodianthrones hypericin and pseudohypericin, and flavonoids such as quercetin, quercitrin, rutin, and hyperoside [65]. Ahn et al. [66] concluded that *H. perforatum* extracts also have the capacity to inhibit the reuptake of dopamine, serotonin, and norepinephrine, improving ADHD symptoms [66]. Table 5 shows the most relevant plant-derived bioactive compounds with neuroprotective effects in ADHD patients.

### 2.3. Neurodegenerative Disorders

Neurodegenerative disorders are defined by the progressive deterioration of neuronal structure and function, leading, eventually, to irreversible loss of neurons and other brain cells [67]. Concurrently, the WHO has suggested that neurodegenerative disorders could represent one of the most prevalent causes of death in developed countries. Neurodegenerative disorders, such as Parkinson’s disease (PAD) and Alzheimer’s disease (AD), are the most prevalent disorders, and they are thought to have the greater impact on public health and represent a heavier economic burden [68]. PD is the most common progressive movement disorder characterized by motor and non-motor symptoms. Conversely, AD is the most common form of dementia, and patients experience symptoms such as memory loss, inability to learn, and deterioration of behavioural function [69]. At present, more than one million people with PAD and 10.46 million AD patients live in Europe, and this number is forecast to double by 2030 [70].

#### 2.3.1. Alzheimer’s Disease: Plant-Based Bioactive Compounds as Therapeutic Agents

AD therapy is focused on the use of cholinesterase inhibitor medicines and NMDA receptor inhibitor. However, therapy is symptomatic, and the response tends to reduce disease progression. Several studies have found plant-based bioactive compounds with neuroprotective properties via (i) acetylcholisterase (AChE) and butyrylcholinesterase (BChE) inhibition, (ii) oxidative stress counteraction using high antioxidant activity, and (iii) neuroinflammation marker protein activity. Several plants, namely Citrus plants, *R. rosea*, *Glutinous rehmannia* (Gaertn.) Steud., *Dracocephalum moldavica* L., *C. sinensis*, *Morinda lucida* Benth, *Glycyrrhiza glabra* L., *Vitis vinifera* L., *Salvia fruticosa* Mill., *Glycine max* (L.) Merr., *Gochnatia polymorpha* (Less.) Cabrera, and *Vaccinium* sp. have been described as a good source of phytocompounds that can be used to help neurodegenerative disorder management.

*Citrus* plants are one of the most important fruit crops, including orange, lemon, lime, grapefruit, and kumquat, and these plants are widely investigated for their bioactive composition and their health benefits. According to Zhi et al. [71], Olayunka et al. [72], and Ahsan et al. [73], some bioactive-derived flavonoid compounds obtained from *Citrus* fruit extract, namely the flavanone naringenin, the flavonol quercetin, and the polymethoxyflavone sinensetin, presented neuroprotective activity [71,72,73]. As demonstrated by Ahsan et al. [73], naringenin demonstrated neuroprotective effects against AB1-42-evoked neurotoxicity by restoring AMPK levels and decreasing AB concentration in mouse neuronal cells, as well as autophagy-inducing ability by alleviating beclin-1 ATG5 and ATG7 in Neuro2a cells and primary mouse neurons [73].

On the other hand, a sinensetin-based bioactive compound has demonstrated to display anti-inflammatory, antioxidant, and antiapoptotic activities against amyloid beta (Aβ25–35)-induced neurotoxicity. The studies carried out by Zhi et al. [70] demonstrated that the application of sinensetin in SH-SYSY5Y cells reduced oxidative stress and inflammation. Olayunka et al. [72] verified that quercetin significantly enhanced the efficacy of donepezil (a cholinesterase inhibitor) by lowering AChE and Aβ1-42 levels and increasing the GSH (endogenous antioxidant) levels, which reduced lipid peroxidation and reversed neuronal damage.

*R. rosea* is a Crassulaceae perennial flowering plant. Around 140 bioactive compounds are present in the subterranean portions of this species, namely phenols, rosavin, rosin, rosarin, organic acids, terpenoids, phenolic acids and their derivatives, flavonoids, anthraquinones, alkaloids, tyrosol, and salidroside. The *R. rosea*-derived rosiridin monoterpene was shown to reduce neuroinflammation and oxidative stress by the ability to inhibit AChE, which promotes the production of endogenous antioxidants such as GSH, MDA, SOD, and caspase [74].

Rehmannioside A and Tilianin are bioactive compounds obtained from *G. rehmannia* and *D. moldavica* extracts. They were also described to possess anti-neurodegenerative and antioxidant advantages by inducing the activation of Nrf2 and ERNk1/2 and CREB and to block the generation of the JNK-, MAPK, p38-, and NF-B inflammatory response [75,76].

Kaempferol can be found in several plants including kale, beans, tea, spinach, and broccoli. Kaempferol isolated from *C. sinensis* extracts has been demonstrated to have the capacity to activate the production of endogenous antioxidants, such as GSH and SOD, which reduce the oxidative stress and consequently present a positive role in the management of the AD [77]. Likewise, marinoid J and morin are, respectively, a phenylethanoid and flavonol that also showed neuroprotective activity by counteracting MDA and reducing the ^•^NO activity while promoting the production of glutathione-peroxidase in the brain [78]. Furthermore, El-Gazar [79] reported that the combination of MK-801, an NMDA receptor blocker, and morin to treat AD has the potential to block dementia-related protein expression, namely Aβ 42, ApoE, tau, and -catenin phosphorylation.

Glycyrrhizic acid, myricetin, resveratrol, and sulforaphane obtained from extracts of *G. glabra*, *V. vinifera*, and species from Cruciferae family (cabbages), respectively, also presented antioxidant activity by blocking ROS generation. In addition, the flavonoids myricetin and dihydromyricetin were demonstrated to have an array of biological effects, namely the facility to suppress the Fe^2+^-induced cell death in SH-SY5Y cells and also anti-AChE activity [80]. Similarly, curcumin also presented antioxidant activity though the ROS generation inhibition [81], whereas the sulforaphane molecular mechanism showed that this bioactive compound promoted the glutathione peroxidase RNA expression and also inhibited the peroxidation and deposition of AB [82].

Rosmarinic acid, coumestrol, ginsenoside, caryophyllene, procyanidin, helichrysoside, and bisabolol obtained from different plants presented similar molecular mechanisms, which include the inhibition of the Aβ aggregation and ROS generation, as well as to block of the monoamine oxidase activation, which led to an increase in antioxidant activity [83,84,85,86,87,88]. Table 6 shows the most relevant plant-derived bioactive compounds with neuroprotective effects in AD patients.

#### 2.3.2. Parkinson’s Disease: Plant-Based Bioactive Compounds as Therapeutic Agents

PAD is a neurodegenerative condition defined by the gradual death of dopaminergic neurons in the brain’s substantia nigra. The buildup and aggregation of misfolded proteins, notably alpha-synuclein, form insoluble clumps known as Lewy bodies in the molecular pathways underlying PAD. This degenerative process results in defective protein breakdown, mitochondrial malfunction, oxidative stress, and neuroinflammation, which eventually leads to neuronal death.

The most often used treatment strategy for PAD is the use of levodopa, a dopamine precursor. Levodopa is turned into dopamine in the brain and helps to reduce motor symptoms like tremors and stiffness by replenishing dopamine levels. Long-term usage of levodopa, on the other hand, may cause motor fluctuations and dyskinesias. Dopamine agonists and anticholinergic are another therapeutic alternative.

Baicalein isolated from the *S. baicalensis* plant can be used to treat PAD by boosting the dopamine and 5-hydroxytryptamine levels in the basal ganglia [89]. A study performed in Hela and SH-SY5Y cells indicated that baicalein reduced the α-synuclein oligomers production as well as their aggregation [89].

Silva et al. [90] verified that ethanolic extracts of the *Erythrina velutina* Willd. plant can scavenge free radicals and reduce the neurotoxicity in by 6-OHDA in SH-SY5Y cells. Furthermore, Jiménez-Cabrera et al. [91] indicated that the *Erythrina* genus produces a wide range of secondary metabolites, including flavanones, isoflavones, isoflavones, and pterocarpans, namely sigmoidin A and B, erycristagallin, abyssinone V-4′-methyl ether, waragalone, mildbenone, 2″-*O*-galloyl orientin, neobavaisoflavone, and hypaphorine, which are responsible for the antioxidant activity of the plant. The beta-carboline alkaloids harmalol, harmaline, and harmine extracted from Peganum harmala can eliminate lipid and protein oxidation in the brain and also the death of dopaminergic neurons [92].

The flavonoids of *Carthamus tinctorius* L. also present antioxidant and anti-inflammatory activities. Kaempferol, hyperoside, naringenin, quercetin, and luteolin isolated from *C. tinctorius* increased the DA transporter and DA levels as well as DJ-1 protein expression [93]. The isoflavone puerarin, isolated from the root of *Pueraria lobata* (Willd.) Sanjappa and Pradeep, inhibited the proteasomal malfunction. Additionally, this isoflavone also lowered the caspase-3 activity and the ratio of bcl-2/bax. Moreover, puerarin also has protective effects in the tyrosine hydroxylase (TH)-positive neurons [93].

The ginsenosides Rb1 and Rg1 are regarded to be the primary molecules responsible for ginseng’s therapeutic properties. As indicated by Rahman et al. [94], ginsenosides are capable of enhancing the antioxidant activity by reducing ROS generation and maintaining the intracellular adenosine triphosphate (ATP) levels. Additionally, ginsenoside Rb1 can disaggregate fibrils and inhibit α-synuclein polymerization [94].

Kiasalari et al. [95] found out that *H. perforatum* extracts decreased the Bax levels, the neuronal damage, and also the apoptotic process.

The monoterpenes bornyl acetate, α-pinene, β-pinene, and δ-terpinene extracted from *C. asiatica* have the ability to inhibit AChE activity [96]. Likewise, the alkaloids berberine, groenlandicine, palmatine, jateorrhizine, coptisine, and epiberberine extracted from the rhizomes of *Coptis chinensis* Franch also have the ability to inhibit AChE. Moreover, groenlandicine and epiberberine also inhibited the beta-secretase enzymatic activity [97]. Table 7 shows the most relevant plant-derived bioactive compounds with neuroprotective effects in PAD.

## 3. Conclusions

Oxidative stress is a major factor in psychiatric, neurodevelopmental, and neurodegenerative diseases. Unfortunately, these pathologies are getting more prevalent over the world. Individual factors such as age, family history, alcohol and smoking consumption, lifestyle, sleep issues, diabetes, cardiovascular disorders, and environmental risk factors, including pollution, climate change, and socioeconomic status, and even the COVID-19 pandemic, all contribute to oxidative stress.

To combat the negative effects of oxidative stress, the human body has evolved various antioxidant mechanisms throughout the centuries. However, the human body’s natural antioxidant defence mechanism is often scarce, requiring exogenous antioxidant supply to scavenge free radicals.

The intake of natural and exogenous antioxidants with neuroprotective effects is a good strategy to reduce oxidative stress and prevent the development of diseases. Here, we present the evidence that several natural products, including vitamin C, *C. sinensis* polyphenols, *H. perforatum*, and *C. sativus*, have shown promise in lowering oxidative stress and treating symptoms of major depressive disorder (MDD). Similarly, bioactive compounds such as curcumin, luteolin, resveratrol, quercetin, and plants like *A. gramineus*, *R. rosea*, and *G. biloba* are associated with neuroprotective effects and symptom improvement in neurodevelopmental disorders such as autism spectrum disorder (ASD) and attention deficit/hyperactivity disorder (ADHD). Furthermore, in neurodegenerative diseases, natural compounds from *R. rosea*, *M. lucida*, and *G. rehmannia* provide neurological improvement. As a result, it makes sense to continue to investigate all these plant-derived bioactive compounds to confirm their efficacy, pharmacological doses, and safety in clinical samples of neuro-psychiatric patients.

## Figures and Tables

**Table 1:** Plant-derived bioactive compounds with neuroprotective actions in MDD patients.

		Neuroprotective-Based Activity					
Plant	Bioactive Compound	Antioxidant	Anti-Inflammatory	Antidepressant	OS Reduction	Increase on BDNF Levels	Refs.
*Citrus* sp.	Ascorbic acid	X					[20]
*Camelia sinensis* (dose 50–100 mg/kg mice)	Catechin, gallocatechin, epicatechin, kaempferol, quercetin, gallic acid, and chlorogenic acid	X	X				[21,22]
*Crocus sativus* (dose 5–10 mg/kg mice)	Curcumin, crocins, crocetin, picrocrocin, and safranal	X	X				[25,26]
*Hypericum perforatum* (dose 0.1–1 mg/kg mice)	Hyperforin, rutin, and melatonin	X	X	X			[23]
*Hypericum triquetrifolium* (dose 0.1–1 mg/kg mice)	Hypericin				X	X	[24]

**Table 2:** Plant-derived bioactive compounds with neuroprotective effects in BD patients.

		Neuroprotective-Based Activity					
Plant	Bioactive Compound	Na Channels Block	GABAergic Block	Kinase C Inhibition	Cholinergic Increase	Improve Antioxidant	Refs.
*Crocus sativus* (dose 0.15–0.35 mL/kg mice)	Safranal; crocin					X	[31]
*Mentha* spp., *Carum carvi* dose 50–100 mg/kg mice)	Carvone	X	X				[32]
Vegetables (dose 50–100 mg/kg animal)	Gallic acid				X	X	[33]
*Cammelia sinensis* (dose 10–40 mg/kg mice)	Quercetin			X		X	[34]

**Table 3:** Plant-derived bioactive compounds with neuroprotective effects in SZ patients.

		Neuroprotective-Based Activity					
Plant	Bioactive Compound	ERbB Stimulation	CNNB-r Inhibition	PHPH Increase	Il-6 Levels Decrease	BDNF Levels Increase	Refs.
*Scopolia carniolica Scopolia japonica*	Scopoletin	X		X			[37]
*Reynoutria japonica*	Emodin	X		X			[38]
*Curcuma longa*	Curcumin				X	X	[39]

CNNB-r—cannabinoid receptors; PHPH—phosphorylation.

**Table 4:** Plant-derived bioactive compounds with neuroprotective effects in ASD patients.

		Neuroprotective-Based Activity					
Plant	Bioactive Compound	OS Reduction	TNF Reduction	MMP-9 Redution	GSH Increase	IL Redution	Refs.
*Curcuma longa* (dose 200 mg/kg autistic rats)	Curcumin	X	X	X	X		[44]
Vegetables/fruits (dose 1 mg/kg mice)	Luteolin	X	X			X	[46,47,48,49]
Grapes, peanuts, cocoa, berries (dose 20–80 mg/kg mice)	Resveratrol	X	X				[51]
Broccoli(dose 50–150 µmol)	Sulforaphane	X			X		[52]
*Matricaria recutita*, *Sophora chrysophylla*, *Camelia sinensis* (dose 75–300 mg/Kg mice)	Quercetin Rutin	X					[53,54]

OS—oxidative stress.

**Table 5:** Plant-derived bioactive compounds with neuroprotective effects in ADHD patients.

		Neuroprotective-Based Activity					
Plant	Bioactive Compound	OS Reduction	AChE Reduction	Il Supression	Dopamine Increase	TrkB, BDNF Reduction	Refs.
*Acorus gramineus* (dose 0.1 mg/kg mice)	α-Asarone, β-asarone	X	X	X	X	X	[58]
*Scutellaria baicalensis* (dose 2–10 mg/kg mice)	Oroxylin A, baicalin	X			X		[59]
*Ginkgo biloba* (dose 50 mg/patient)	Ginsenoside				X	X	[60]
*Pinus pinaster* (dose 25 mg or 50 mg/patient)	Pycnogenol	X			X		[61]
*Curcuma longa* (dose 1 g/kg mice)	Curcumin, Curcuminoid	X					[62]
*Camelia sinensis* (dose 2 mg/kg rats)	Catechin, EGCG				X		[63]

AChE—acetylcholinesterase activity; Il—interleukin; EGCG—epigallocatechin gallate.

**Table 6:** Plant-derived bioactive compounds with neuroprotective effects in AD patients.

		Neuroprotective-Based Activity					
Plant	Bioactive Compound	NrF Activation	Antioxidant	Antiapoptotic	OS Reduction	AChE Decrease	Refs.
*Citrus* plants (dose 50 µM/cells culture)	Naringenin, quercetin, sinensetin		X		X		[71,72,73]
*Orthosiphon stamineus* (dose 10/20/40 µM/SH-SY5Y cell)	Sinensetin		X	X			[72]
*Cammelia sinensis* (dose 12.5–25 mg/kg mice)	Quercetin		X		X	X	[74]
*Rhodiola rosea* (dose 1 mg/kg mice)	Rosidin		X		X	X	[74]
*Glutinous rehmannia* *Dracocephalum moldavica* (dose 80 mg/kg mice)	Rehrmannioside A, tilianin	X	X				[75,76]
*Cammelia sinensis* (dose 10 mg/kg mice)	Kaempferol,		X		X		[77]
*Morinda lucida* (dose 5 mg/kg mice)	Morin, marinoid J		X		X		[78,79]
*Curcuma longa* (dose 1–15 µM/SH-Sy5Y cells)	Curcumin		X		X		[81]
*Elaeagnus glabra* f. *oxyphylla*	Procyanindin						[84]
*Glycine max* (dose 40–160 µM/kg mice)	Coumestrol		X		X		[85]
*Aralia nudicaulis* (dose 2.5 µM/mouse neuroblastoma neuro-2a)	Ginsenoside		X		X		[87]
*Gochnatia polymorpha* (dose 1–1000 mouse neuroblastoma neuro-2a)/NSC-34 cells)	Bisalol		X		X		[88]

**Table 7:** Plant-derived bioactive compounds with neuroprotective effects in PAD.

		Neuroprotective-Based Activity					
Plant	Bioactive Compound	Reduction α-Synuclein	OS Reduction	Antioxidant	DA Levels	Proteasomal Activation	Refs.
*Scutellaria baicalensis* (dose 25–100 µM/SH-Sy5Y cell culture)	Baicalein						[89]
*Erythrina velutina* (dose 25 µg/mL/SH-SY5Y cell culture)	Sigmoidin A and B, erycristagallin, abyssinone V-4′-methyl ether, waragalone, mildbenone, 2″-O-galloyl orientin, neobavaisoflavone, and hypaphorine		X	X			[90]
*Peganum harmala* (dose 300–600 mg/kg mice)	Harmalol, harmaline, and harmine		X		X		[92]
*Carthamus tinctorius* (dose 10 mg/kg rats)	Kaempferol, hyperoside, naringenin, quercetin, and luteolin			X	X		[93]
*Pueraria lobata* (dose 25 or 50 mg/kg rats)	Puerarin		X	X			[94]
*Ginkgo biloba*	Ginsenosides	X	X	X			[95]
*Hypericum perforatum* (dose 200 mg/kg rats)	Naphthodianthrones and phloroglucinols		X	X			[96]
